# Identifying resource-conscious and low-carbon agricultural development pathways through land use modelling

**DOI:** 10.1016/j.landusepol.2024.107208

**Published:** 2024-08

**Authors:** Aniket Deo, Paresh B. Shirsath, Pramod K. Aggarwal

**Affiliations:** Borlaug Institute for South Asia (BISA), International Maize and Wheat Improvement Centre (CIMMYT), New Delhi 1100012, India

**Keywords:** Land Use Modelling, Resource management, Low-carbon Pathways, Spatial Planning

## Abstract

Increasing agricultural production with current resources and technology may lead to increased GHG emissions. Additionally, large population countries like India face substantial challenges in terms of food demand, agro-ecological heterogeneity, carbon footprint and depleting natural resources, thus increasing the decision complexities for policymakers and planners. We aim to examine the potential of producing more food from available agricultural land with low-carbon (reduced GHG emissions) and resource-conscious (optimal resource use) options. The current study develops multiple calorie production and emission-centric land use using a land use optimization model wherein the calorie production and emission objective, resource and emissions constraints, and food production targets interact across multiple spatial levels. The capabilities of the developed model are demonstrated with a case study in India targeting ten crops (grown over two seasons) covering three food groups (cereals, legumes, and oilseeds). Three hypothetical scenarios for each objective of maximizing calories production (*Calories-nation*, *Calories-group*, *Calories-crop*) and minimizing GHG emissions (*Emissions-nation*, *Emissions-group*, *Emissions-crop*) are developed concerning targets of national crop production (*Calories-nation*, *Emissions-nation*), state food groups production (*Calories-group*, *Emissions-group*), and state crop production(*Calories-crop*, *Emissions-crop*), with different spatial levels of constraints. A maximum growth of 11% in calorie production is observed in *Calories-nation* while mitigating 2.5% emissions. Besides, the highest emission reduction of around 30% is observed in *Emissions-group* but with no change in calorie production. Emission scenarios can spare up to 14.8% land and 18.2% water, while calorie production-maximization scenarios can spare a maximum of 4.7% land and 6.5% water. The optimization-based methodology identifies the regions of altered land use by proposing appropriate crop substitution strategies, such as increasing oilseeds in Rajasthan and soybean in east Maharashtra. Many states show conservative production growth and emission reduction with state-level crop production targets (*Calories-crop*), suggesting crop redistribution within the state alone will not be sufficient unless improved technologies are introduced. The maximum growth and mitigation potential estimated in this study may be affected by climate shocks; therefore, introducing the improved technologies needs to be coupled with a crop redistribution mechanism to design climate-resilient and futuristic land use systems. The proposed land use model can be modified to incorporate climate change effects through consideration of scenarios of changed crop yields or through direct/indirect coupling with dynamic crop simulation models.

## Highlights

• Calories and emissions-centric land use options explored through optimization.
• Six scenarios of two objectives & spatially differential constraints formulated.
• 11% growth in calorie production and 30% GHG reduction could be achieved in India.
• Optimal crop redistribution at multi-scales proposed within current resource base.

## 1 Introduction

The increasing global population has increased food demands and resulted in significant pressure on finite land resources and global warming (He et al., 2022). As per the Paris agreement (2015), there is a strong emphasis towards reducing greenhouse gas (GHG) emissions by 43% by 2030 to limit global warming within 1.5°C (Anderson and Peters, 2016) as a goal towards carbon neutrality. However, to meet the growing food demands, the GHG emissions from agricultural production systems increased by 30% in past two decades(Wang et al., 2021).

Agricultural land use contributes significantly to GHG emissions primarily through production practices of crops and livestock (Wu et al., 2022) which release gases such as carbon-dioxide (CO_2_), methane (CH_4_) and nitrous oxide (N_2_O). An increase in concentration of GHG leads to greenhouse effect, resulting in global warming and climate change (Montzka et al., 2011).

Carbon neutrality goals are proposed by many countries for example “*double carbon target*” by China (Zhao et al., 2023), “*A European Green Deal in Brussels*” by EU (Wu et al., 2022) and “*carbon-neutral society by 2050*” by Denmark (Wu et al., 2022). These goals emphasize on reducing carbon emissions as an integral pathway for carbon neutrality (Chuai et al., 2022, Wu et al., 2022). Reducing GHG emissions can take place through both preventing emissions and removing emissions from the atmosphere (McCarl and Schneider, 2001, McCarl and Schneider, 2000).

Land use alterations can significantly affect GHG emissions (Chuai et al., 2022) which also provides an opportunity to design agricultural land use that reduces GHG as acknowledged by many researchers such as (Roe et al., 2021, Smith et al., 2013). However, land-based food production systems are under pressure to meet growing food demands (Koh et al., 2013) with limited arable land, water, labor, energy, and budget (Peltonen-Sainio et al., 2019). Moreover, the agricultural resources are competing with urbanization (Su et al., 2014) and other sectors (manufacturing, service etc). Therefore, there is a need to examine the potential of producing more food from agricultural land use while reducing GHG emissions and sparing already scarce agricultural resources. The current study focuses on exploring resource-conscious and low-carbon food production options. In the context of current study, “resource-conscious” means optimal utilization of available resources, while “low-carbon” signifies reduction in GHG emissions (including CO_2_ emissions), resulting in judicious resource use and a low-carbon pathway.

Different geographies are suitable for producing certain crops, but their actual production or yield potential largely depends on non-limiting supply of water, nutrients, pest management, labor and knowledge (Evans, 1996, Evans and Fischer, 1999). Since actual production is the product of both crop area and actual yield, an increase in either or both these factors is desirable for increase in production. But many researchers agree to avoid significant expansion of croplands mainly because of preservation of natural habitats and biodiversity (Lobell et al., 2009) and rather call for sparing of cropland (Lal, 2018, Schneider et al., 2022). On the other hand, increasing yield or reducing yield gap pose challenges of limited access to resources, lack of knowledge and technology and inadequate infrastructure (Snyder et al., 2017).

An alternate approach is the spatial redistribution of crops among suitable geographies to enhance yields (Wang et al., 2022) and production while adhering to emission targets and current resource availabilities (Davis et al., 2019, Davis et al., 2017). The approach of crop redistribution coupled with emission targets and resource optimization has proven to generate efficient land use policies and is well accepted in the domain of land use planning (LUP) (Memmah et al., 2015, Rajni et al., 2018, Wang et al., 2022). A general approach of spatial crop redistribution-based LUP studies that aim to reduce GHG emissions includes estimation of crop statistics including production and resource requirement data at spatial scale and GHG emissions incurred through crop management practices. Many of them use an optimization algorithm with emissions and economic targets to process the dataset subject to certain constraints and conditions. Wang et al. (2022) developed an optimization model which integrated crop redistribution and improved management for producing sufficient food with lower GHG emissions in China. Davis et al. (2019) proposed spatial redistribution of cereals crops in India using an optimization framework to minimize GHG emissions and resource-use with nutrition and economic targets and constraints. Tang and Hailu (2020) did a farm-scale crop land optimization in China to study changes in farm profitability and production-induced GHG emissions. Moreover, other relevant studies covering spatial LUP have also included environmental and demographic pressures (Liu et al., 2013), advanced management practices (Klein et al., 2013), food-water-energy-carbon nexus (Chamas et al., 2021), demand-side interventions (Shirsath and Aggarwal, 2021), and climate-smart interventions (Dunnett et al., 2018, Shirsath et al., 2017) in regional LUP.

The current study addresses a critical and timely issue that has global relevance, namely the intersection of land use alteration, food production and climate change mitigation. We use a unique perspective and practical approach to develop multiple production and emission-centric land use using a simplistic land use optimization model. Unlike many other studies, our study focuses on realized yield at district-level which has an advantage in terms of more realistic planning. Realized yield is the yield achieved by farmers at the production level with current resources and technology base such as land, irrigation, capital, and management practices. This yield varies significantly over time and space due to different physical and biological factors, as well as differences in crop management practices between regions. The variability in yield allows the solution space for crop redistributions.

The study combines production and emission objectives, resource and emissions constraints, and food production targets across multiple spatial levels. This holistic integration addresses the complex interactions that exist in the real world, allowing us to develop more effective land use plans. In this study, we attempt a novelty in conceptualizing a linear programming model formulation which can develop appropriate land use options and we check whether production levels could be increased, GHG emissions be reduced and agricultural resources such as land, water, labor and budget could be spared with the current technology and current resource base. We apply our modelling framework to a case study in India, a country facing substantial challenges in terms of food demand, agro-ecological heterogeneity, carbon footprint and depleting natural resources. Ten crops (grown over two seasons) covering three food groups (cereals, legumes, and oilseeds) are targeted. Three hypothetical scenarios for each objective of maximizing calorie production (*calorie-nation, calorie-group, calorie-crop*) and minimizing emissions (*emissions-nation, emissions-group, emissions-crop*) are developed concerning national-level food targets (*calories-nation*, *emissions-nation*), state-level targets of food groups (*calories-group, emissions-group*) and state-level targets with the individual crop (*calories-crop, emissions-crop*) with different spatial levels of constraints. To the best of our knowledge, such conceptualization of spatial land use modelling in the context of India has not been done in the existing literature.

## 2 Material and methods

### 2.1 Study area and data requirements

**Fig. 1:**
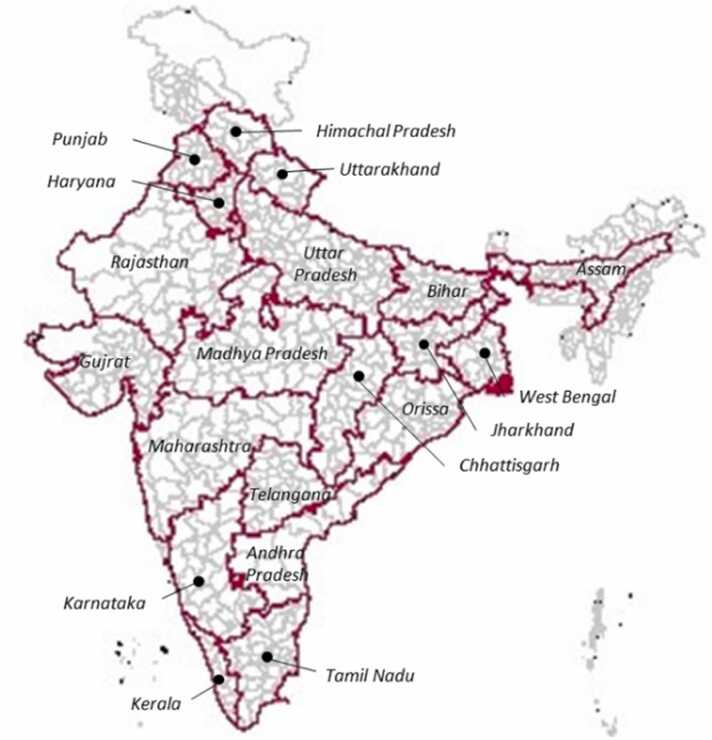
The study area map showing selected states for the current study (bold red outline). The polygons in grey show the district boundary.

India is the seventh largest country in the world with an area of 328.8 million hectares out of which around 50% of land is arable (Pathak et al., 2022). In this study, we considered 20 major agricultural states (589 districts) which cover around 87% of the country’s geographical area (Fig. 1).

This study primarily needs crop production statistics, cost of cultivation, and constraints databases at different scales on land, water, capital. Ten major crops were selected for the study (rice, maize, wheat, sorghum, pearl millet, pigeon pea, groundnut, mustard, soybean, and chickpea) which cover around 133 million hectares and constitute almost two-third gross sown area in the country (MoAFW, 2021). The ten crops considered here are primarily food crops produced for the human diet, except for maize (Ray et al., 2022). Maize is used largely for feed (animal/poultry), and only 13% is used as food (IIMR, 2024). Although the share of major crops in the human diet is only 29% globally, India has a large vegetarian population and has a higher share (70%) of these crops in the diet (Ray et al., 2022).

Rice, maize and sorghum are grown substantially in two seasons (Monsoon and winter) therefore the total commodities in this study were 13 considering 7 single season crops and 3 double season crops. The baseline land use consisting of above crops produce around 325.2 million tons food, 510 million Megajoules (MJ) calories and agricultural income of around 46 billion USD with 41.2 billion USD and 753 billion m^3^ of capital and irrigation requirement respectively. Besides, the baseline production is responsible for generating 177 million tons of GHG emissions.

The summary production statistics of these crops, their respective food group and the season are provided in Table 1. Table 1 also lists the standard deviation of yield, cost of cultivation, irrigation requirement and emissions which reflect the spatial variability present in India. This variability across India highlights the scope for crop redistribution within suitable geographies.

**Table 1:** Summary statistics of the crops considered for the analysis in the current study. Superscript *, # and ^ represent cereal, oilseed and legume crop respectively. The values in parentheses are standard deviations.

**Crops**	**Total Area** **(M ha)**	**Total Production** **(M t)**	**Yield** **(t/ha)**	**Cost of cultivation**^a^ **(USD/ha)**	**Irrigation requirement** **(mm/ha)**	**Emissions** **(kgCo**_**2eq**_**/ha)**	**Calories** **(KJ/100 gm)**
Monsoon Rice*	40.0	103.5	2.53 (± 0.91)	295 (± 122)	689 (± 163)	2765 (± 1347)	1527
Winter Wheat*	32.4	120.2	3.14 (± 1.14)	321 (± 82)	606 (± 119)	1154 (± 175)	1515
Monsoon Soybean^#^	11.4	11.4	1.02 (± 0.43)	273 (± 85)	447 (± 58)	442 (± 41)	1870
Winter Chickpea^	9.6	10.9	1.21 (± 0.39)	285 (± 88)	427 (± 59)	420 (± 145)	1580
Monsoon Maize*	7.2	20.3	2.86 (± 1.53)	328 (± 169)	462 (± 72)	803 (± 258)	1530
Monsoon Pearl Millet*	7.1	8.9	1.52 (± 0.7)	166 (± 57)	287 (± 63)	557 (± 117)	1456
Winter Mustard^#^	6.0	8.8	1.13 (± 0.51)	212 (± 74)	452 (± 55)	710 (± 128)	2132
Winter Rice*	4.3	15.9	3.34 (± 0.7)	407 (± 113)	911 (± 150)	2765 (± 1347)	1527
Monsoon Groundnut^#^	4.0	7.0	1.63 (± 0.69)	600 (± 260)	471 (± 111)	591 (± 67)	2390
Winter Sorghum*	3.4	2.8	1.2 (± 0.97)	310 (± 223)	642 (± 58)	521 (± 97)	1380
Monsoon Pigeon pea^	3.9	3.3	0.96 (± 0.38)	298 (± 122)	381 (± 76)	363 (± 94)	1440
Winter Maize*	2.1	10.6	4.66 (± 2.3)	500 (± 198)	646 (± 95)	803 (± 258)	1530
Monsoon Sorghum*	1.9	2.0	1.23 (± 0.67)	266 (± 86)	379 (± 78)	521 (± 97)	1380

a 1 USD ≈ 82 INR

The agricultural production dataset from 2017 to 2019 containing district-level yields, cropped area and production of selected crops was obtained from the Directorate of Economics and Statistics (DES), Department of Agriculture and Farmer Welfare, Government of India. The DES aggregates village level area and production to derive district-level yields. Similarly, aggregation of district-level data produces state and national data. With the DES data, variability or standard deviation can be estimated at state and national level.

Additionally, state-level cost of cultivation data and harvest prices were also obtained from the DES. The total cost of cultivation included costs of seeds, human labour, machine labour, irrigation, fertilizers, FYM, biocides and miscellaneous (10% of operational costs) costs (Shirsath et al., 2017). We used gridded rainfall (0.25 degrees) and temperature (1 degrees) data from India Meteorological Department, Government of India (Pai et al., 2014). Calorie content of the selected crops is obtained from sources such as (Longvah et al., 2017) and U.S. Department of Agriculture's (USDA).

### 2.2 Methodological framework

**Fig. 2:**
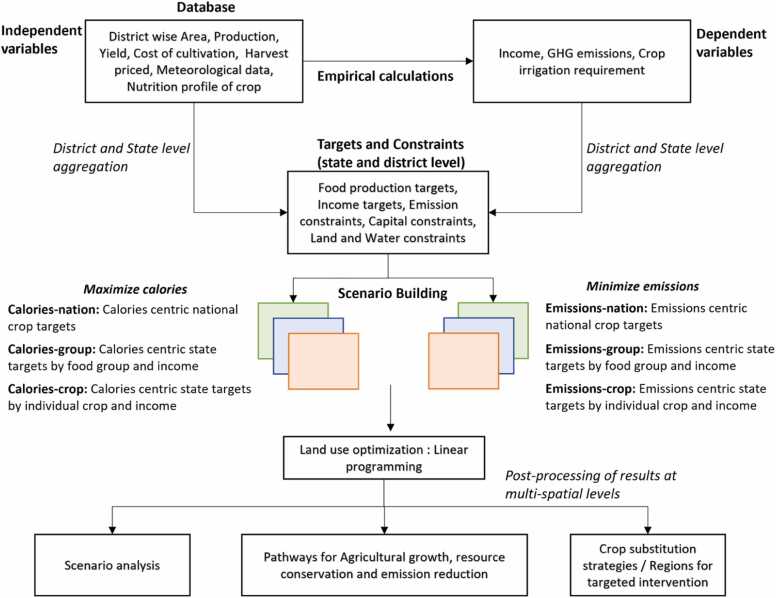
**:** Methodological framework of the study.

Fig. 2 illustrates the methodological framework used to generate required input data and develop land use options. As the initial step, district-level average values of crop area, production, yield, rainfall, and temperature for the year 2017–2019 are extracted from the dataset. These independent variables are used to derive dependent variables such as GHG emissions, irrigation requirements and income using empirical relations. The GHG emissions for crops are estimated using The Cool Farm Tool (Hillier et al., 2011) which is an emission calculator that employs a life cycle assessment approach and integrates data from land use, crop management and emission factors. The crop irrigation requirement is calculated using climatic water balance method wherein we used Hargreaves method (Hargreaves et al., 1985) for reference evapotranspiration (ETo), FAO-56 single crop coefficient method (Allen et al., 1998) for Crop ET and USDA-SCS method (Dastane, 1978) for effective rainfall calculations. An irrigation efficiency of 60% is considered in this study (Brouwer et al., 1988). Hargreaves method (Hargreaves et al., 1985) is an empirical approach to estimate reference evapotranspiration which is usually used when availability of meteorological data is limited. FAO-56 single crop coefficient approach (Allen et al., 1998) is a multiplicative relationship of crop specific coefficients and reference evapotranspiration to produce crop water requirement. USDA-SCS (Dastane, 1978) is a method to estimate the portion of rainfall that contributes to runoff and soil erosion. It involves adjusting the total rainfall by considering various factors that affect runoff, such as soil type, land cover, slope and conservation practices. The above empirical methods are applied to derive data at district scale as demonstrated by Shirsath et al. (2017).

The crop level data is aggregated at district and state levels to produce land, water, emissions and capital constraints and production and income targets. We use the term “target” to refer to the current baseline values, which represent the existing state of agriculture in the studied states and districts. These baseline values act as reference points for our scenario analysis. Baseline land availability, production and the income are derived from production statistics and their production-cost-benefit calculations are used as production and income targets. The cost of cultivation and harvest price is used to derive the agricultural income at district-level. The cost of cultivation and the baseline crop areas are used to estimate the current capital requirement which is considered as agricultural budget requirement. Similarly, the crop irrigation requirement and baseline crop areas give the net irrigation requirement at district-level. These baseline capital and water requirements also suggest the current availability of capital and water hence these baseline values are considered as upper limit or constraints. Current GHG emissions are estimated and used as upper limit. The targets and constraints vary by scenarios details of which are described in the next section. A linear programming model using simplex method (*linprog* function) in MATLAB 2016b is used for land use optimization wherein the land use options are generated for the six scenarios. The result from optimization is processed to produce a comparison of the scenarios, the crop redistribution/substitution strategies, patterns for targeted interventions and regional growth potential.

#### 2.2.1 Scenario Building and Formulation

For this study, six scenarios are developed to understand the changes in land use options when objectives, constraints and targets are applied differently. For this study, six scenarios are developed to understand the changes in land use options when objectives, constraints and targets are applied differently. These six scenarios represent growth pathways for low-carbon, resource-conscious development, which could be applied at multi-scale (state/national). The design of different scenarios considers multiple policy goals (food security, emission reduction, land, and water sparing). These scenarios also consider spatially differential resource availability, which is likely to be the case during future development.

Land and water being immovable resources are limited to any change. Therefore, the model land use policy shall adhere to the land and water conditions at the district-level. Thus, the land and water constraint remain same for all the scenarios. Similarly, as agricultural budget is primarily a state subject in India (Reddy, 2017), the capital constraint is applied at the state-level. Following is the description of scenarios:

Calories-centric national crop targets (Calories-nation): The scenario maximizes the national calorie production and considers a crop wise production target at national level. No state-level baselines are used. Eq. 1 represents the objective function of *Calories-nation* and (2), (3) represent the land and water constraints at district-level respectively. Eq. 4 represents emission constraints at state-level while Eq. 5 represents the crop-wise production target at national level.

(1) $$Z_{\max}=\sum_{j=1}^{m}\sum_{i=1}^{n}\left(Y_{\mathrm{ij}}\times {\mathrm{Cal}}_{i}\right).X_{\mathrm{ij}}$$

(2) $$\sum_{i=1}^{n}X_{\mathrm{ij}}\leq L_{j}$$

(3) $$\sum_{i=1}^{n}X_{\mathrm{ij}}.w_{\mathrm{ij}}\leq W_{j}$$

(4) $$\sum_{j=1}^{o}\sum_{i=1}^{n}X_{\mathrm{ij}}.e_{\mathrm{ij}}\geq E_{k}$$

(5) $$\sum_{j=1}^{m}\sum_{i=1}^{n}X_{\mathrm{ij}}.Y_{\mathrm{ij}}\geq P_{i}$$

Where, *m* and *n* are the number of districts in India and number of crops considered in this study respectively such that j=1:589 and i=1:13. X_ij_ is the decision variable and represents the area (ha) allotted to i^th^ crop in j^th^ district. In Eq. 1, Y_ij_ represents the yield (t/ha) of i^th^ crop in j^th^ district while Cal_i_ represents the calorie content (Kcal/t) of i^th^ crop. Z_max_ is the objective function that aims to maximize the total calorie production by aggregating calories produced in 589 districts. In equation 2, L_j_ represents the land available in j^th^ district while in Eq. 3, w_ij_ and W_j_ represent the water requirement (mm) of i^th^ crop in j^th^ district and water available (m^3^) in j^th^ district. In Eq. 4, index *o* is the number of districts in k^th^ state, e_ij_ is the emission of i^th^ crop in j^th^ district and E_k_ is the emission limit in k^th^ state. In equation 5, P_i_ represents the national level production (t) of i^th^ crop.

Calories-centric state targets by food group and income (Calories-group): This scenario uses the same objective function of *Calories-nation* but considers a capital constraint and an income target, both applicable at state-level. The production target is scaled down from national level to state-level however instead of crop-wise target in *Calories-nation*, *Calories-crop, Calories-group* uses a food group-wise target at state-level. This means all the cereals crops such as rice, wheat, maize, sorghum, and pearl millet are grouped together to achieve a total cereal target irrespective of the crop-wise contribution. A similar strategy is used for legumes and oilseeds. The production target of a food group is an aggregation of baseline values of all the crops in a food group. *Calories-group* uses Eq. 1, 2, 3 and 4 from *Calories-nation*. The capital constraint, income target and food group wise target at state-level are represented by Eqs. 6, 7 and 8 respectively.

(6) $$\sum_{j=1}^{o}\sum_{i=1}^{n}X_{\mathrm{ij}}.\;C_{\mathrm{ij}}\leq B_{k}$$

(7) $$\sum_{j=1}^{o}\sum_{i=1}^{n}{(X}_{\mathrm{ij}}.\;Y_{\mathrm{ij}})\times \mathrm{Mij}-{(X}_{\mathrm{ij}}.\;C_{\mathrm{ij}})\geq I_{k}$$

(8) $$\sum_{j=1}^{o}\sum_{i=1}^{p.}X_{\mathrm{ij}}.\;Y_{\mathrm{ij}}\geq P_{g}$$

Where, *o* and *p* are the number of districts in k^th^ state and number of crops in g^th^ food group wherein g=1:3. In equation 6, B_k_ represents the agricultural budget of k^th^ state where k=1:20. In Eq. 7, M_ij_ is the harvest price of i^th^ crop in j^th^ district while I_k_ represents the Income target in k^th^ state. In equation 8, P_g_ represents the state-level production target of g^th^ food group.

Calories-centric state targets by individual crop and income (Calories-crop): The scenario is similar to *Calories-group* but largely differs in the production targets. The food group-wise target in *Calories-group* is replaced with, crop-wise production targets at state-level in *Calories-crop*. This means the model land use should adhere to the state budget and attain at least the state baseline income and production targets. The equations of objective function, land, water, capital constraints and income target (Eq. 1, 2, 3, 4, 6 and 7) remain the same for *Calories-crop*. Eq. 9 represents the crop wise production target at state-level.

(9) $$\sum_{j=1}^{o}\sum_{i=1}^{n}X_{\mathrm{ij}}.\;Y_{\mathrm{ij}}\geq P_{k}$$

Where, P_k_ is the production target in k^th^ state in Eq. 9.

Emission-centric national crop targets (Emissions-nation): The scenario minimizes national level emissions as an objective while following other conditions exactly same as *Calories-nation*. *Emissions-nation* uses (2), (3), (4), (5) from *Calories-nation* while emission minimization objective represented by Eq. 10 is used.

(10) $$Z_{\min}=\sum_{j=1}^{m}\sum_{i=1}^{n}\left(e_{\mathrm{ij}}\right).X_{\mathrm{ij}}$$

Emission-centric state targets by food group and income (Emissions-group): Eq. 10 is used as objective while other conditions are like *Calories-group*. Eqs. 2, 3, 4, 6, 7 and 8 are used from *Calories-nation* and *Calories-group*.

Emission-centric state targets by individual crop and income (Emissions-crop): This scenario uses Eq. 10 as objective while other conditions are similar to *Calories-crop*. It takes Eqs. 2, 3, 4, 6, 7 and 9 from *Calories-nation* and *Calories-crop*.

Table 2 presents a summary of the scenario conditions discussed above.

**Table 2:** Scenario-wise objective, constraints, and their respective levels.

**Scenario name**	**Objective function at national level**	**Constraints**	**Spatial Level**
Calories-centric national crop targets (*Calories-nation*)	Maximize calories	Land, water	District
		Emissions	State
		Crop production targets	National
Calories-centric state targets by food group and income (*Calories-group*)	Maximize calories	Land, water	District
		Income targets, Emissions, Capital	State
		Cereals, legumes, and oilseed production targets	State
Calories-centric state targets by individual crop and income (*Calories-crop*)	Maximize calories	Land, water	District
		Crop production targets, Income targets, Emissions, Capital	State
Emission-centric national crop targets (*Emissions-nation*)	Minimizing emissions	Land, water	District
		Emissions	State
		Crop production targets	National
Emission-centric state targets by food group and income (*Emissions-group*)	Minimizing emissions	Land, water	District
		Income targets, Emissions, Capital	State
		Cereals, legumes, and oilseed production targets	State
Emission-centric state targets by individual crop and income (*Emissions-crop*)	Minimizing emissions	Land, water	District
		Crop production targets, Income targets, Emissions, Capital	State

To check the model's capabilities to replicate the current conditions, we tested it using rigid model boundaries. Along with Land and water constraints, crop-wise production targets, income targets, emissions and capital constraints were set at the district-level. As expected, the model generated a similar land use as the baseline, which confirms that the model formulation is accurate (data not shown).

### 2.3 Sensitivity analysis

A sensitivity analysis is done by varying the input parameters such as crop irrigation requirements, capital availability and crop emissions to understand the changes in calorie production and net emissions. The current study doesn’t incorporate a dynamic pricing model therefore the changes in the market prices are beyond the scope of current study. A 5%, 10% and 15% reduction in crop irrigation requirement (LHS Eq. 3) is considered in sensitivity analysis while the total available water at district-level (RHS Eq. 3) remains constant. For sensitivity analysis. the capital availability is increased by 5%, 10% and 15% (RHS Eq. 6) which could be achieved by providing more budget to agricultural sector. Lastly, the crop emissions are reduced by 5%, 10% and 15% (LHS Eq. 4) while the emission limit (RHS Eq. 4) is constant. The range of variation considered in the sensitivity analysis is realistic as several water and emission smart techniques have shown saving of 7%-92% (Touil et al., 2022) and about 75% (Pathak et al., 2010) respectively.

## 3 Results

The current study explores the potential of improvement in calories production, emission reduction, agricultural income and land and water sparing with six scenarios. Here, land and water sparing means redistributing crops to produce more or adequate food on less land and reduced irrigation demand respectively.

### 3.1 Trade-off in performance parameters and resource sparing

**Fig. 3:**
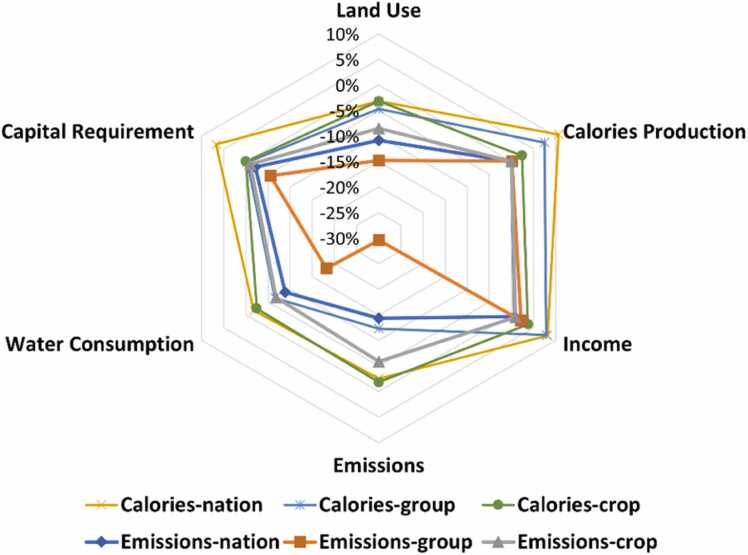
Percent change in performance parameter for different scenarios.

Fig. 3 shows the potential of trade-offs in performance parameters (land, water, capital, income, emission) for different scenarios as compared with the baseline levels. The two themes namely calories maximization and emission minimization produced land use with distinct characteristics. The emission-centric scenarios had larger potential of agricultural land and water sparing while fulfilling the production targets while calories centric scenarios produced substantial growth in calories production and agricultural income as evident in Fig. 3. The maximum potential of 30% in emission reduction was observed in *Emissions-group* without any growth in calories production, however this led to sparing of capital (5.5%), land (15%) and water (1%) resources and minor growth in income (2.2%). Maximum growth of 11% in calories production was observed in *Calories-nation* with an emission reduction potential of 2.5% but with 6.7% higher capital requirement. The maximum land and water sparing potential of 15% and 18% respectively was observed in *Emissions-group* in addition to growth of 2% in income and 5.5% capital saving. With state-level capital constraints, the growth in calories production in *Calories-group* and *Calories-crop* is restricted. Moreover, with state-level crop targets, the change potential drastically reduces as observed in *Calories-crop* and *Emissions-crop*. However, state-level food group-wise targets allow space for crop substitution within the food group thus enabling substitution of high yielding crops (*Calories-group*) and low emission crops (*Emissions-group*) over others in the food group.

In all the scenarios, land and water constraints are applicable at district-level since they are immovable resources. In *Calories-nation*, the maximum production at district-level could be attained by utilizing either land or water resource completely. A crop redistribution within a district could allow exhaustion of land yet spare water or vice versa. In *Calories-group* and *Calories-crop*, it may happen that land or water or both resources are not utilized completely due to state-level capital constraints. For example, there are 13.7% districts (mostly in Tamil Nadu, Bihar, Uttar Pradesh) in *Calories-group* where land and water resources remain underutilized. Around 19% and 21% of the districts show water savings of at least 5% in Monsoon and Winter season respectively in *Calories-group*. While in *Calories-nation*, around 8.5% and 10% districts show at least 5% water saving potential in Monsoon and Winter season, respectively. This supports the observation from Fig. 3 that the water sparing potential of *Calories-group* is higher than *Calories-nation*. It is interesting to note that *Calories-nation* enables Winter water sparing in Southeast Maharashtra which is one of the most drought prone areas in the country. This is because of shift towards low water intensive crops such as substitution of winter sorghum with chickpea. Moreover, *Calories-nation* allows maximum land sparing of 11% in Karnataka followed by 9% and 7% in Rajasthan and Andhra Pradesh respectively. In terms of water sparing, *Calories-group* allows higher water sparing potential with maximum sparing of 9% in Andhra Pradesh followed by 5% in Maharashtra and Andhra Pradesh.

In terms of emission-centric scenarios, a potential of upto 15% and 18% land and water sparing exists respectively, because the objective minimizes emissions by allocating just sufficient land that would fulfil the production targets. Therefore, growth in calorie production is not observed in any of the emission-centric scenarios. There are around 32% districts in *Emissions-nation* and *Emissions-group* where both land and water are spared. Around 50% of the states show land and water sparing potential of at least 10% in *Emissions-nation* and *Emissions-group* with maximum sparing of land and water being 46% and 64% in Tamil Nadu in *Emissions-group* respectively. The resource sparing at state-level is governed by crop redistribution which is discussed in the following section.

### 3.2 Land use change

**Fig. 4:**
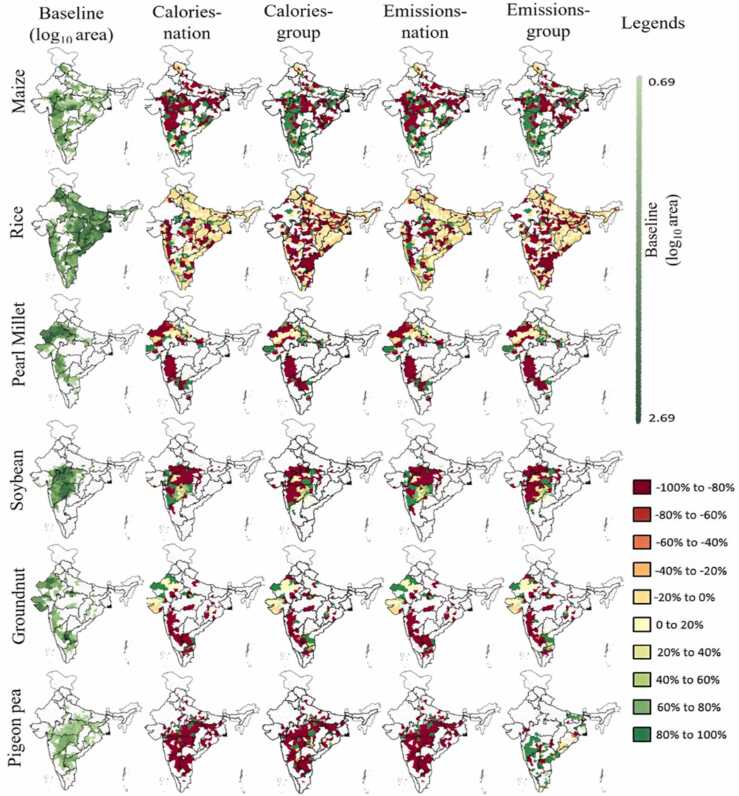
Baseline area and land use changes for Monsoon crops at district-level are shown as percent change in *Calories-nation*, *Calories-group*, *Emissions-nation* and *Emissions-group* with respective to baseline area. The white space represent that the crop is currently not produced in that district. Change outside the range −100%< c > 100% is truncated to −100% and 100% respectively. *Calories-crop* and *Emissions-crop* are not shown due to insignificant changes in land use.

Considerable land use change occurs in *Calories-nation*, *Calories-group*, *Emissions-nation* and *Emissions-group* that justifies the significant changes in their performance parameters (Fig. 3). Scenario *Calories-crop* and *Emissions-crop* show trivial land use change therefore they are not discussed here and not represented in Fig. 4 which displays spatial land use change. On an aggregate basis, monsoon and winter maize area is increased in calories-centric scenarios (*Calories-nation* and *Calories-group*) and *Emissions-group* while reducing acreages of other crops (Table 3). In addition to maize, the aggregate area under mustard is increased in *Calories-group*. Unlike any other scenario, the national areas under winter sorghum, pigeon pea and ground nut area are increased in *Emissions-group*. In case of *Emissions-nation*, the national level area of all crop decrease. A closer look at district scale suggest that the Monsoon maize area is reduced in several district as seen in Fig. 4 yet, the aggregate change remains positive in *Calories-nation*, *Calories-group* and *Emissions-group* as several large district show increase in maize area. Adhering to the objective function, the model favours high calorie crop or low emission crop for substitution, but the calorie being a function of yield and emissions depending on geography vary in districts. Therefore, as seen for *Calories-nation* in Fig. 4, the rice and maize area in many districts are reduced and substituted with the respective highest calories crop (e.g., soybean in Maharashtra). In *Calories-nation* and *Emissions-nation*, a clear pattern of increase in area for soybean and groundnut (Fig. 4) is visible in Maharashtra and Rajasthan, respectively. A reduction in pearl millet acreage (*Calories-nation* and *Emissions-nation*) is found in most districts of Maharashtra and Rajasthan which is substituted with soybean and groundnut, respectively. In general, the geographic trend of land use change in *Calories-nation* -*Emissions-nation* and *Calories-group*-*Emissions-group* appear to be similar largely because of similar system constraints and targets (Section 2.2.1) but their magnitude of change remains distinct.

**Table 3:** Land use at national level for Baseline and other scenarios. The values in parentheses are the percent change w.r.t to baseline. Superscript * and # represent monsoon and winter crop respectively.

**Crop**	**Baseline area, M ha**	***Calories-nation***	***Calories-group***	***Calories-crop***	***Emissions-nation***	***Emissions -group***	***Emissions -crop***
Rice*	40.0	38.7 (-3)	32.1 (-20)	39.3 (-2)	38 (-5)	21.4 (-47)	38.2 (-5)
Wheat^#^	32.4	31.7 (-2)	29.5 (-9)	32.1 (-1)	31.2 (-4)	28.4 (-12)	31.1 (-4)
Soybean*	11.4	10.8 (-5)	8.8 (-23)	10.6 (-7)	10.3 (-9)	8.9 (-22)	10.5 (-8)
Chickpea^#^	9.6	9 (-7)	8.2 (-15)	8.9 (-8)	9.5 (-1)	7.4 (-23)	8.9 (-8)
Maize*	7.2	11.5 (59)	18 (149)	7.9 (9)	5.3 (-27)	16.8 (132)	6.2 (-14)
Pearl Millet*	7.1	5.8 (-19)	6.1 (-14)	6.9 (-3)	5 (-30)	4.5 (-37)	5.4 (-24)
Mustard^#^	6.0	5.5 (-9)	7.3 (21)	5.9 (-2)	5.7 (-5)	5.5 (-9)	5.5 (-9)
Rice^#^	4.3	4.3 (0)	1.7 (-60)	4.3 (0)	4.2 (-2)	1 (-77)	4.2 (-2)
Groundnut*	4.0	3.2 (-20)	3.2 (-20)	3.7 (-7)	3.3 (-17)	4.1 (3)	3.7 (-7)
Pigeon pea*	3.9	2.3 (-41)	3.6 (-7)	2.9 (-25)	2.6 (-33)	4.5 (16)	2.8 (-28)
Sorghum^#^	3.4	1.4 (-59)	0 (-100)	2.4 (-30)	1.4 (-59)	0.2 (-94)	2.2 (-36)
Maize^#^	2.1	4.5 (118)	8.4 (307)	2.4 (16)	1.4 (-32)	8.8 (327)	1.7 (-18)
Sorghum*	1.9	0.9 (-52)	0 (-100)	1.7 (-9)	0.9 (-52)	2.2 (18)	1.3 (-30)

**Fig. 5:**
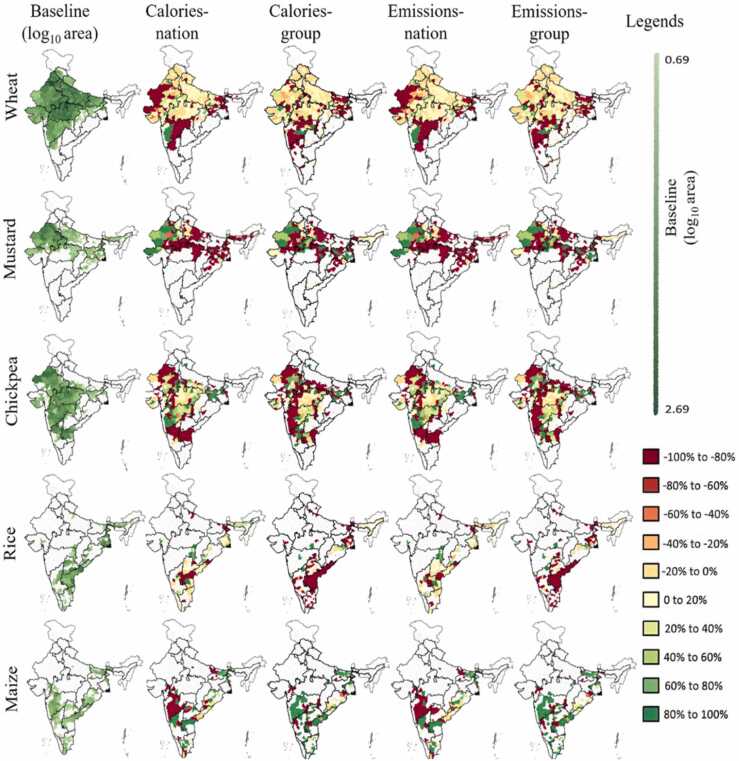
Baseline area and land use changes for Winter crops at district-level are shown as percent change in *Calories-nation*, *Calories-group*, *Emissions-nation* and *Emissions-group* with respective to baseline area. The white space represent that the crop is currently not produced in that district. Change outside the range −100%< c > 100% is truncated to −100% and 100% respectively. *Calories-crop* and *Emissions-crop* are not shown due to insignificant changes in land use.

In case of Winter season (Fig. 5), the wheat dominant regions such as central India show minor change in *Calories-nation* and *Emissions-nation* however, wheat area in west Gujrat and Rajasthan is substituted with Mustard (Fig. 5). But several districts in central India show reduced area in Mustard thus the aggregate change in Mustard remain negative in *Calories-nation* and *Emissions-nation* (Table 3). Similar to Monsoon maize, the maize in Winter season shows reduced area in several districts but increase in large districts therefore the aggregate change remains positive. The land use in *Calories-nation* can meet the national production target and produce 11% surplus yet spare around 4.5 Mha of land.

In *Calories-group* and *Emissions-group*, a reduction in Monsoon rice, pearl millet, soybean and groundnut areas in visible in most districts while these crops are majorly substituted with Maize. A major growth in maize area in *Calories-group* and *Emissions-group* is observed in the western part of the country specifically in west Maharashtra and Madhya Pradesh (Fig. 5) while several districts in central and east region experience reduction in maize area. Even though there is substantial increase in maize area, yet rice remains the dominant crop (considering both seasons) in cereals for all scenarios. As seen in Fig. 4, the trend for all scenarios in pearl millet remains the same as its area in Rajasthan is substituted with groundnut. In Winter season of *Calories-group* and *Emissions-group*, a net reduction in wheat, winter rice and chickpea appear (Fig. 5). Like Monsoon season, winter maize gets substituted with these crops. The is no land allocated to Winter sorghum (Table 3) in *Calories-group* which is substituted with Winter maize.

In *Calories-group*, national cereal area is reduced by 1% while its production is increased by 9%. This is because of redistribution of crops among the cereal food group and localised expansion of high calorie crops at favourable geographies as seen in Fig. 4 and Fig. 5. In legumes and oilseeds, there is no increase in production however the baseline targets are met by conserving 13% and 10% land, respectively in *Calories-group*. The reduction of legumes and oilseeds in several districts is apparent in Fig. 4 and Fig. 5.

A commonality is observed in all scenarios with respect to oilseeds wherein the area of ground nut and mustard is increased in Gujrat and Rajasthan which are already top oilseed producing states. Soybean area is reduced in most of the districts, yet it remains the dominant crop in Oilseeds. In general, the land use analysis suggests that the dominant crops in baseline scenario remains prevailing even in the model scenarios which is seen at an aggregate national level in Table 3.

**Fig. 6:**
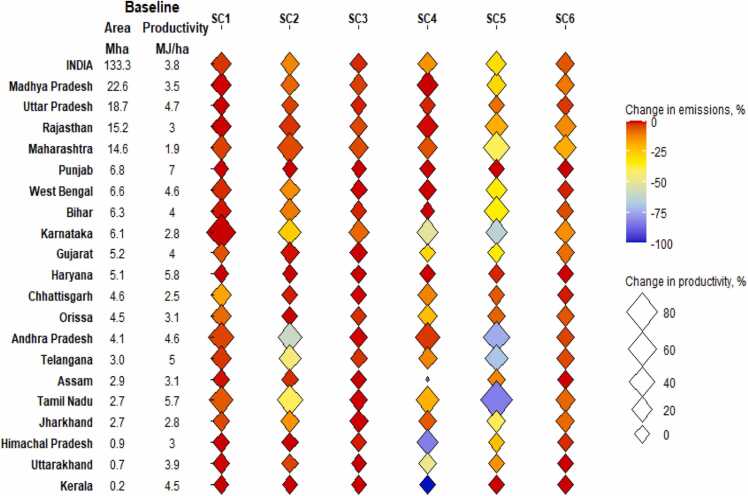
Change in land productivity and emissions across states and scenarios. States are arranged in decreasing order of baseline landuse area.SC1: *Calories-nation*, SC2: *Calories-group*, SC3: *Calories-crop*, SC4: *Emissions-nation*, SC5: *Emissions-group*, SC6: *Emissions-crop*.

Land productivity is an important performance indicator that suggest the potential of a land to produce calories. A crop redistribution within the state would alter the cropping intensity hence affecting the land productivity and environmental impacts. In this study, land productivity refers to calories production per unit area. Fig. 6 shows the potential of increasing the land productivity and change in GHG emissions in different states of India. Fig. 6 also indicate the baseline land use and land productivity of states suggesting that Madhya Pradesh is the largest agricultural state while Punjab is the most productive. In *Calories-nation*, around 60% of the states show at least 10% growth in land productivity however, the emission reduction is negligible in most states accept Chhattisgarh which show 17% reduction. Among the calorie-centric scenarios, only *Calories-group* show simultaneous growth in land productivity and substantial emission reduction. The emission-centric scenarios show significant change in emissions principally because of its objective of emission minimization. Assam and Himachal Pradesh show at least 80% reduction in *Emissions-nation* while Andhra Pradesh and Tamil Nadu show at least 75% reduction in *Emissions-group*. It could be observed in Fig. 6 that both *Emissions-nation* and *Emissions-group* enable growth in land productivity as well as emission reduction in most states. Even without growth in calorie production, the land productivity increases because the state land use reduces while maintaining the production targets.

It is interesting to note that highly productive states such as Punjab and Haryana show negligible change in productivity and emission reduction in all scenarios while Tamil Nadu which is another high productivity state show substantial changes. Even in *Calories-crop*, states such as Karnataka, Maharashtra and Rajasthan show at least 8% growth in productivity and at least 4% emission reduction. Similarly, in *Emissions-crop*, above states show at least 19% growth in productivity and at least 4% emission reduction. Even with strict boundary conditions in *Calories-crop* and *Emissions-crop*, above states show substantial change suggesting that these states have more scope of crop redistribution to fulfil its production targets, mitigate emissions and contribute to national food production.

It is worth noting that states such as Maharashtra and Telangana show a higher productivity growth in *Calories-group* and *Emissions-group* than *Calories-nation* and *Emissions-nation* respectively despite *Calories-group* and *Emissions-group* having state-level production targets. In Maharashtra, the model favours Maize in *Calories-group* and *Emissions-group* but Soybean in *Calories-nation* and *Emissions-nation* (Fig. 4 and Fig. 5) which results in larger calories production in *Calories-group* (due to high yield calorie product of maize). Similarly, in Telangana, the increase in maize area is higher in *Calories-group* than *Calories-nation*. The crop substitutions change the contribution of states towards national production but even then, for most crops the top contributing states remain the same such as Uttar Pradesh for rice and wheat, Rajasthan for pearl millet and mustard, Madhya Pradesh for maize and Gujrat for groundnut.

### 3.3 Sensitivity analysis

Among the three variables (crop irrigation requirements, capital availability and crop emissions) studied in sensitivity analysis, the capital availability was most sensitive followed by crop irrigation requirement and then crop emissions. When capital availability was increased, *calories-group and emission-group* scenarios showed variation up to positive 5% and negative 3.7% change in calorie production and net emissions respectively. As *calorie-nation* and *emission-nation* scenarios do not have any capital constraint, therefore their sensitivity to capital availability not applicable. For sensitivity of crop irrigation requirement, *calories-nation* and *emission-nation* showed variation up to positive 4.5% and negative 2.3% change in calorie production and net emissions respectively. However, other scenarios showed trivial changes suggesting that even with water saving technologies these scenarios would not benefit significantly. The sensitivity of reducing crop emissions showed minor changes of up to positive 0.37% and negative 0.9% in calorie production and net emissions respectively for scenarios *calories-nation* and *emission-nation.* For other scenarios, the changes in calorie production and net emissions were insignificant when crop emission was varied. *Calories-crop* and *emissions-crop* scenarios were studied in sensitivity analysis but not discussed here because negligible variations were observed.

## 4 Discussion

### 4.1 Spatial planning for resource management

Agricultural land use has a major impact on land, water, regional economics and GHG emissions (Hasan et al., 2020). With reducing agricultural resources bases and global or regional commitments towards mitigation goals, land use planning becomes necessary. Spatial planning enables regional structuring of land use to balance the demand, resource consumption and impacts on environment (Yoshida et al., 2020). The current study generates effective land use plans which minimize agricultural resource use while producing more or at par with baseline conditions. The suggested crop redistribution strategies are derived from optimization of resources at local level such that the regional objectives and targets are met while increasing the local resource use efficiency. An increase in state-level land productivity (Fig. 6) implies improvement in resource efficiency as more calories are produced in fewer land. Moreover, an increase in land productivity is accompanied by emission reduction in many states (*Calories-group*, *Emissions-nation*, *Emissions-group*) which emphasizes that the proposed options of land use are resource-conscious and low-carbon. The spared resources could be used efficiently to generate carbon sinks such as grasslands, forests etc. or systematic urban infrastructure. The proposed model framework operates at multiple spatial levels by using national/state-level objectives which interact with constraints and targets at variable national/state and district levels. The ability of the framework to consider 589 districts with 13 crops and 2 seasons makes it competent for national level planning.

The results of this study are in general agreement with a similar nationwide study for India done by Devineni et al. (2022) which highlighted that the government’s procurement targets can be met by shifting the geographies where crops are grown while increasing net farm income and arresting groundwater depletion. For food production maximization pathways. this study suggested that there is less scope for crop redistribution in Punjab and Haryana, while Tamil Nadu shows a large potential for crop redistribution. Similarly, in emission-centric scenarios, crop redistribution presents significant potential for mitigating emissions, meeting production targets, and conserving land and water resources substantially. All these scenarios highlight land sparing with varying magnitude. However, we did not analyze whether the spared land would be suitable to generate carbon sinks or be indeed conserved or it would be put to non-agricultural use. All spared land was cropland in our model formulation, and the redistribution evaluated here considers only the ten food crops; no other crops/vegetation of the region were considered in this analysis. There is a large competitive demand for spare land, especially from non-agricultural sectors. Few emerging renewable energy interventions, such as Agri-photovoltaics, complement agricultural land use. Regional variation in land sparing depends on the objective and constraints defining the trajectory through scenario analysis. The current analysis provides information on possibilities of land-sparing using multiple scenarios; this analysis does not evaluate the likelihood of this land is actually spared or put to other use, which is not considered here.

The results of this study, especially for highly productive areas of North-western India, show less scope for crop redistribution and, hence, land sparing. Increased productivity in this region has not been translated to land-sparing largely because of the counteracting policies on guaranteeing minimum support price and assured procurement of rice and wheat.

### 4.2 Low-carbon agricultural growth pathways

For a sustainable future, the agricultural growth should harmonize with environment (Shirsath and Aggarwal, 2021). The current study provides an understanding of calorie, resources (water-land-capital) and emission-centric land use as pathways for agricultural growth. The calorie-centric scenario enables growth in production (+11%) without surpassing the baseline emissions. Essentially, the crops are redistributed in a manner that high yielding regions are encouraged to grow more, which increases the net regional production. However, the crop redistribution complies with emission constraints therefore the redistribution favors high-yield and low-emissions regions. Conversely, the emission-centric scenarios reduce the emissions while meeting the production targets. Here, the crop redistribution strategy produces just-sufficient calories by redirecting resources to high-yielding regions. By this, the production targets are met with reduced land, water and capital resources which results in reduced emissions. The crop substitution strategies proposed in this study for different scenarios guide a plausible course of action to address the challenges of agricultural emissions, food production and sustainable land use development. This substitution has been evaluated here from the supply-side/production perspective for the ten major crops considered in the current analysis. The aspects of demand and pricing have not been evaluated, which would need either linkage with General/Partial Equilibrium Models or the design of detailed pricing modules. The crop substitution or diversification identified here has been studied earlier (Bhogal and Vatta, 2021, Rakshit et al., 2021), and few policies or incentives have been launched by governments on crop substitution mainly to conserve groundwater resources (GoI, 2014) and to reduce the environmental footprints (GHG emissions as well air pollution) (Priyadarshini and Abhilash, 2020).

A similar analysis for Bihar, India done by Shirsath and Aggarwal (2021) did quantification of trade-offs between food production, emissions, and income under technology and food demand-shift scenario and climate change. This study demonstrated that under different technology growth pathways, both food self-sufficiency and reduction in emission intensity could be achieved if we relax constraints on dietary demand and focus on production (kilo-calorie) maximization targets. For the current resources and climate, the findings of this study are in congruence with Shirsath and Aggarwal (2021) and can provide guidance to achieve India’s long-term low-carbon development strategy (MoEFCC, 2022).

### 4.3 Food production and emissions tradeoffs

Reducing agricultural emissions is challenging since the reductions achievable by changing farming practices are limited (MacLeod et al., 2010, Franks and Hadingham, 2012). Agricultural emissions reduction as a pathway for carbon neutrality depends on choosing low-carbon land use as per the carrying capacity and food demands (Röling, 1997). Recently researchers have identified several cost-effective opportunities for climate change mitigation in Indian agriculture (Dunnett et al., 2018, Khatri-Chhetri et al., 2023, Sapkota et al., 2019, Sapkota et al., 2018). But transitioning into low-carbon land use may not suffice growing food demands or income with traditional technologies. On the other hand, high-yield land use may not be carbon and resource-conscious (Valin et al., 2013). The interrelation between agricultural production, emissions and resources remains complicated, and there exist tradeoffs (Valin et al. (2013); Chamas et al. (2021); Shirsath and Aggarwal 2021). The current study identifies solution space for the conflicting objective of calorie maximization and emission minimization. The production-centric land use types developed in this study increase calorie production without surpassing baseline emissions while emission-centric land use types substantially mitigate emissions and fulfill baseline production targets. Both the objective themes have advantages and disadvantages therefore the identification of the tradeoffs provides an understanding of the limits of production, resource requirements and GHG emissions. Increasing land productivity is an efficient method to increase food availability and reduce emission pressure (Valin et al., 2013). However, high-production technologies incur cost, and their inaccessibility remains a major issue in developing countries. The current study proposed crop redistribution based on realized yield as a medium to increase land productivity which could minimize the tradeoffs between food security (production), emissions and resource requirements.

The findings of the current study indicates that crop substitution can be an effective option for reducing emissions without compromising on food production and in agreement with (Lamb et al., 2016) who found that a land-sparing strategy has the technical potential to achieve emission reductions from agriculture in UK.

### 4.4 Land use policy

Agricultural land use changes are influenced by demand side-push and urbanization across agro-climatic zones in India (Mittal and Hariharan, 2016). Policy interventions related to land use change incur significant costs and impacts therefore informed decision making is crucial (Dunnett et al., 2018). For example, the state mandate to delay the transplanting date of rice in Punjab to conserve groundwater left the farmers with less time to prepare for next crop and lead them for stubble burning as a quick resort causing tremendous pollution (Balwinder-Singh et al., 2020). Usually, the scope of agricultural growth is unevenly spread in the country (Davis et al., 2019) and advancing crop management practices alone may not lead to sustainable agricultural growth (Tesfaye et al., 2017) A probable strategy for low-carbon regional food security and well acknowledged by researchers is shifting of crops to suitable areas (Davis et al., 2017; Dunnett et al., (2018a, 2018b); Shirsath et al., 2017). Wang et al. (2022) analyzed crop redistribution and management strategies for China. They noted that meeting the increased food demand and decreasing environmental costs would require the combination of strategies (crop distribution, improved management) leading to increased productivity and decreased required farm inputs and environmental costs.

This study evaluates crop substitution as a key strategy where management is reflected through realized yields. Key take away from the current study is that to increase food production and income with existing resources it is necessary to allow land use change and crop substitution across the states as per crop suitability which could produce up to 11% and 8% growth in calories production and income respectively. Besides, production growth is also possible with food group wise targets even if the targets are set at state-level like in *Calories-group* which can achieve upto 7.5% and 7.8% growth in production and income. On the contrary, crop substitution across the states allows emission reduction (*Emissions-nation*) but maximum reduction of upto 30% occur with food group wise state targets (*Emissions-group*). Moreover, in calories-centric scenarios, there is simultaneous growth in calorie production and reduction of emissions (by at least 2%). Conversely, in emission-centric scenarios, emissions reduce without any concurrent increase in calorie production.

The targeted interventions (crop redistribution) vary across the states with certain states experiencing significant benefits (Fig. 6). For example, Tamil Nadu shows large potential of crop distribution while high yielding states like Punjab and Haryana have limited scope of crop distribution.

The local markets trends influence production patterns which could be captured by state crop targets. However relaxing state crop targets and allowing crop substitution across the states could enable maximum growth potential in calorie-centric scenario as suggested in this study. With advancement in agricultural supply chains in India (De and Singh, 2021), the inter-state trade has become efficient therefore crop substitution across the states would not affect the local dietary patterns. While, in emission-centric scenarios, food group-wise state production target leads to large reduction in emissions than crop-wise state production targets. The results highlight the trade-off among various scenarios which would improve the understanding of policy makers and support them in designing localized and targeted interventions. Future climate changes are mostly negatively impacting crop suitability and yields and changing the pest and disease dynamics through altered interactions between crops, insect pests and their natural predators (Aggarwal et al., 2022). Land use policies need to make a balance by ensuring food security and sustainability under future climatic uncertainties; few studies have demonstrated the utility of land use models (Dunnett et al., 2018, Hinz et al., 2020). The land use tool described here can be modified to incorporate climate change effects through consideration of scenarios of changed crop yields or through direct/indirect coupling with dynamic crop simulation models.

An integrated approach is necessary to implement land use strategies (Karki et al., 2023) to minimize the tradeoffs between food security, socio-economic and environmental goals. Besides the lack of an integrated approach, insufficient funding, inadequate incentive mechanisms, and lack of political will are the key challenges in implementing the land use policies. Nearly free electricity and subsidized fertilizers counteract the goals of crop diversification in well-established rice-wheat cropping systems like in Punjab and Haryana states (Pujara and Shahid, 2016, Saran et al., 2013).

### 4.5 Capabilities and limitations of the study

The development of conceptual framework in this study provides a medium to explore upper limits of agricultural production, resource requirements and environmental impacts. The framework can also be used for conservation planning by setting resource conservation targets, however this is beyond the scope of current study. The framework could be used to understand the tradeoff by interplaying the constraint limits. Additionally, this would enable a sensitivity analysis which will determine the relationship between resources and output.

The model could be implemented elsewhere with similar challenges, however, availability of required data in other parts of the globe is unknown. Therefore, this data-driven approach is only applicable to regions that could produce substantial agricultural data. Another challenge to such national level studies is the dimension of data as large input and output datasets are handled. The current framework is static and deterministic therefore the uncertainty in climate, yields, harvest prices and resource availability are not captured in this study. The current model formulation at this stage ignores the crop substitution effects of food for human diet vs animal feed. The majority of crops considered here, except maize, are primarily food crops and have a large share in Indian diets (Ray et al., 2022). The current modelling framework is flexible enough to incorporate these components and could be attempted in future research.

The current model was used to answer what best could be done from current resources while future foods demand, climate change scenarios and technology interventions were not considered here. The current static nature of the model formulation ignores dynamic factors such as population growth, climate, and economic changes, as well as technological advancements, which hinder the model's ability to provide realistic solutions for the future. However, the scenario and sensitivity analysis could overcome some of these challenges with varying targets and constraints. The implementation of model suggested land use could incur several social and institutional challenges such as local food preferences, farmers heuristics, political will, market logistics, limited funding etc. Pathways to land use changes or crop substitution are not obvious, and they face stiff resistance to change in very well-established cropping systems (e.g., rice and wheat in Punjab/Haryana) where the ecosystem to grow and market these commodities had been well established. Hence, land use policies have been most difficult to implement and have largely remained unsuccessful without market demands, government incentives and political will.

The current model formulation uses realized yields as a baseline, which to some extent manifests into higher use efficiencies and lower emission intensities, not necessarily total GHG emissions. The inefficiencies in low-yield areas may exist because of unused resource use potential, which has not been considered here. The model formulation is able to find pathways by conserving the resources relative to baseline. One limitation of the current formulation is the targets and constraints applied on multiple level depending on the scenario which could add some uncertainties in the analysis. Evaluating the sustainability of resource use needs detailed hydrological-biological linkages which is beyond the scope of the current study; however, the crop substitution here identifies pathways towards lower resource use than the current level without affecting food production or income. The inefficiencies in the current agricultural production systems exist, and these are evident from the yield gap studies (Billen et al., 2024, Zhang et al., 2022). The current model formulation considers the current production levels but is flexible enough to consider the yield gap closures through technological interventions shown by Dunnett et al. (2018).

## 5 Conclusion

The current study provides a simple framework for resource-conscious and low-carbon land use planning for identification of the potential growth in production and reduction in GHG emissions using the current levels of resources and technologies. The landuse optimization methodology combines biophysical datasets which characterize current agricultural production processes and their dynamics. An illustration of the use of the land use modelling framework and databases is demonstrated for India as case study; this illustration drawn the following conclusions:

a) With the current resources, technology base and objective of calorie maximization, the highest growth of around 11% can be achieved in calorie production. This level of growth could be attained by allowing crop redistribution across the state and with a higher agricultural budget than baseline. This would ensure that crop production remains at least at national baseline level while there’s around 2–3% sparing of land and water resources and at the same time achieving reduction of around 2.5% in the GHG emissions. Localized expansion of respective high calorie crops at district-level helps to attain this growth.
b) The maximum emission reduction of around 30% could be attained if food-group wise state targets are set with an objective of emission minimization. This scenario would ensure baseline food production and around 15% and 18% sparing of land and water resources respectively. A balance of low-carbon and high-calorie crops in suitable regions would attain both GHG mitigation and resource sparing, for example increasing the soybean area in Rajasthan by 20% by reducing Kharif rice area.
c) With state-level food group-wise targets, the calorie requirements of the state could be met and at the same time achieve significant growth in production at national level. State-level crop production targets limit the scope of crop redistribution therefore these scenarios produce relatively small growth in production and small reduction in emissions.
d) As the demand for food continues to increase, this study suggests that there is only limited potential for growth in maximum production to meet future food requirements with current agricultural technologies. To address this challenge, it is crucial to consider and prioritize the adoption of newer technologies that can conserve resources and enhance overall production and income.
e) The crop substitution strategy in the current study shows ways to increase food production and income with existing resources, which can be supplemented with an analysis of futuristic what-if scenarios which can guide in locating future growth pathways to achieve food production with lower emissions and optimal resource use.
f) Implementing crop substitutions can encounter challenges such as resistance to change traditional cropping patterns, lack of market infrastructure, coordination among states, and ensuring equitable distribution of benefits. Farmers may hesitate to switch crops due to familiarity, expertise, or financial risks. Overcoming these challenges necessitates careful planning, stakeholder engagement, policy support, and investments in infrastructure and capacity building. Addressing these challenges proactively can lead to successful implementation and positive outcomes for agriculture and food security.
g) The proposed study could be treated as the foundation for future studies to develop more robust models that could consider future food demands, climate scenarios and integrate such optimization models with advanced technological interventions.

## CRediT authorship contribution statement

**Aniket Deo:** Writing – original draft, Software, Methodology, Data curation. **Paresh Bhaskar Shirsath:** Writing – review & editing, Visualization, Data curation, Conceptualization. **Pramod K Aggarwal:** Writing – review & editing, Supervision, Conceptualization.

## Declaration of Competing Interest

The authors declare that they have no known competing financial interests or personal relationships that could have appeared to influence the work reported in this paper.

## Data Availability

Data will be made available on request.
